# Use of machine learning on clinical questionnaires data to support the diagnostic classification of Attention DeficitHyperactivity Disorder: a personalized medicine approach

**DOI:** 10.1192/j.eurpsy.2022.441

**Published:** 2022-09-01

**Authors:** S. Grazioli, M. Mauri, E. Rosi, F. Villa, F. Tizzoni, A. Tarabelloni, S. Trabattoni, V. Mauri, P. Colombo, M. Molteni, M. Nobile

**Affiliations:** Scientific Institute Eugenio Medea, Associazione La Nostra Famiglia, Developmental Psychopathology Lab, Bosisio Parini (LC), Italy

**Keywords:** machine learning, Personalized medicine, Attention Deficit Hyperactivity Disoder, Diagnostic classification

## Abstract

**Introduction:**

Attention Deficit / Hyperactivity Disorder (ADHD) is a highly prevalent neurodevelopmental condition characterized by inattention, motor hyperactivity and impulsivity. ADHD cognitive and behavioral presentation is characterized by a high heterogeneity (APA, 2013). Indeed, a complex diagnostic process, that considers several validated tools, is, to date, necessary.

**Objectives:**

The main aim is to develop supervised machine learning (ML) algorithms that could be used to support the diagnostic process for ADHD, by identifying the most relevant features in discriminating between the presence or absence of the ADHD diagnosis in children.

**Methods:**

We analyzed data from 342 children (Mean age: 8y 8m ± 1y; 61 F) referred for possible ADHD symptomatology. Assessments were performed by an expert clinician and through questionnaires: Social Responsiveness Scale (SRS), Child Behavior Checklist (CBCL), Conners Rating Scale for Parents (CPRS) and for Teachers (CTRS). Data were analyzed using a decision tree classifier and random forest algorithms.

**Results:**

The decision tree model performed an accuracy of 0.71. The random forest model that was identified as the best tested, performed an accuracy of 0.77 (Figure 1) and it identified as most informative parent- and teacher-rated DSM-oriented ADHD symptoms (Figure 2).

**Figure:**
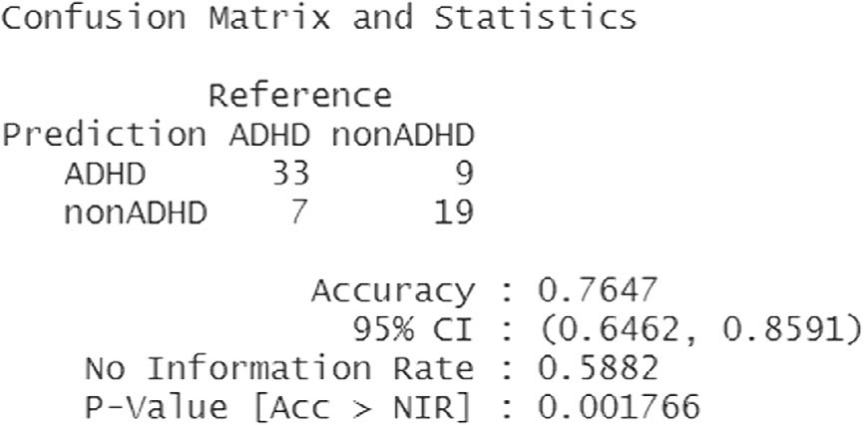

Figure 1: Random forest confusion matrix and statistics.

**Figure:**
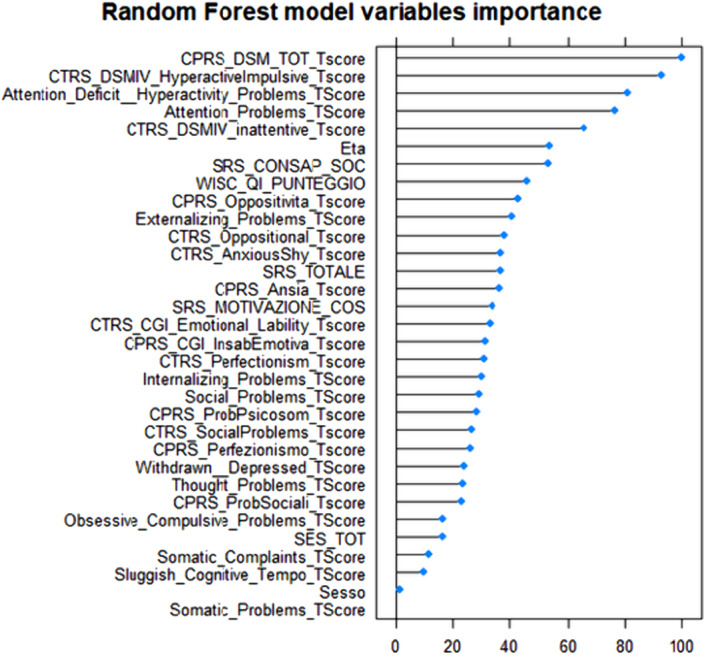

Figure 2: Ranking of variables importance.

**Conclusions:**

A random forest classifier could represent an effective algorithm to support the identification of ADHD children and to simplify the diagnostic process as an initial step. The use of supervised machine learning algorithms could be useful in helping the diagnostic process, highlighting the importance of a personalized medicine approach.

**Disclosure:**

No significant relationships.

